# Achieving a “Grand Convergence” in Global Health: Modeling the Technical Inputs, Costs, and Impacts from 2016 to 2030

**DOI:** 10.1371/journal.pone.0140092

**Published:** 2015-10-09

**Authors:** Colin F. Boyle, Carol Levin, Arian Hatefi, Solange Madriz, Nicole Santos

**Affiliations:** 1 University of California San Francisco, San Francisco, CA, United States of America; 2 University of Washington, Department of Global Health, Seattle, WA, United States of America; US Army Engineer Research and Development Center, UNITED STATES

## Abstract

**Background:**

The Commission on Investing in Health published its report, GlobalHealth2035, in 2013, estimating an investment case for a grand convergence in health outcomes globally. In support of the drafting of the Sustainable Development Goals (SDGs), we estimate what the grand convergence investment case might achieve—and what investment would be required—by 2030.

**Methods and Findings:**

Our projection focuses on a sub-set of low-income (LIC) or lower-middle-income countries (LMIC). We start with a country-based (bottom-up) analysis of the costs and impact of scaling up reproductive, maternal, and child health tools, and select HIV and malaria interventions. We then incorporate global (top-down) analyses of the costs and impacts of scaling up existing tools for tuberculosis, additional HIV interventions, the costs to strengthen health systems, and the costs and benefits from scaling up new health interventions over the time horizon of this forecast. These data are then allocated to individual countries to provide an aggregate projection of potential cost and impact at the country level. Finally, incremental costs of R&D for low-income economies and the costs of addressing NTDs are added to provide a global total cost estimate of the investment scenario.

**Results:**

Compared with a constant coverage scenario, there would be more than 60 million deaths averted in LIC and 70 million deaths averted in LMIC between 2016 and 2030. For the years 2015, 2020, 2025, and 2030, the incremental costs of convergence in LIC would be (US billion) $24.3, $21.8, $24.7, and $27, respectively; in LMIC, the incremental costs would be (US billion) $34.75, $38.9, $48.7, and $56.3, respectively.

**Conclusion:**

Key health outcomes in low- and low-middle income countries can significantly converge with those of wealthier countries by 2030, and the notion of a “grand convergence” may serve as a unifying theme for health indicators in the SDGs.

## Introduction

Human health can be divided into two distinct historical phases. The *first phase* was marked by high rates of infant and young child mortality, and yielded mean life expectancies of less than 40 years. Globally, *Homo sapiens* was a high-mortality, high-fertility species, with relatively modest differences in health outcomes across geographies and communities [1,2]. The *second phase* in human health started around the beginning of the 18^th^ century. Greater wealth from industrialization and trade, improvements in agricultural productivity, advances in science and education, improvements in infrastructure, and an initial wave of public health interventions enabled wealthier populations to increase their life expectancy. Infant mortality declined sharply in the industrializing world. Additional health advances led to subsequent declines in mortality rates among older generations. These improvements, however, primarily benefited richer economies and communities. Poorer nations saw their health outcomes improve, but at a much slower rate than their wealthier peers, leading to a great divergence in global health. And in wealthier economies, significant disparities in health persisted between different socioeconomic groups.

We are still in this second phase, one marked by a sharp divergence of health outcomes between rich and poor nations and communities. The World Bank estimates that average life expectancy in Sub-Saharan Africa in 2012 was just 56 years, and under-5 mortality was 97.6 per 1000 live births [3]. These figures contrast with life expectancy of 80 years and an under-5 mortality rate of just 5.5 deaths per 1000 live births in high income countries. While a range of efforts and commitments by international and domestic players have yielded significant progress in global health over the last 20 years, a substantial burden of preventable mortality and morbidity persists in low-income countries.

In 2013, the Lancet Commission on Investing in Health (CIH) addressed the question of whether the world could enter a *third phase* of human health—one in which poorer countries would see their infectious, maternal, and child health outcomes converge with the levels of wealthier nations—through increased investments in health interventions and systems to combat common causes of mortality and morbidity [4]. In recent years, a number of investment cases have been developed to address the costs of (i) fighting specific infectious diseases, such as HIV/AIDS, malaria, tuberculosis (TB), diarrhoea and pneumonia [5–8]; (ii) rolling out specific categories of health interventions, such as immunization or nutrition [9,10]; (iii) supporting continued innovation in health technologies for low-income nations [11]; (iv) targeting specific vulnerable populations, such as mothers and young children [12–14]; or certain geographic regions [15]; and (v) scaling up innovative financing for health systems to achieve the Millennium Development Goals (MDGs) [16]. While these analyses have all addressed important individual questions, none focused specifically on whether a global convergence in health outcomes could be achieved by investing comprehensively across a very broad range of health conditions. Building upon these existing models, the CIH collaborated with many international agencies and institutions to take an *integrated approach across multiple conditions* to determine if such a grand convergence in health–“to reduce infectious, child and maternal mortality to low levels universally”–was possible and what it might cost.

**Fig 1:**
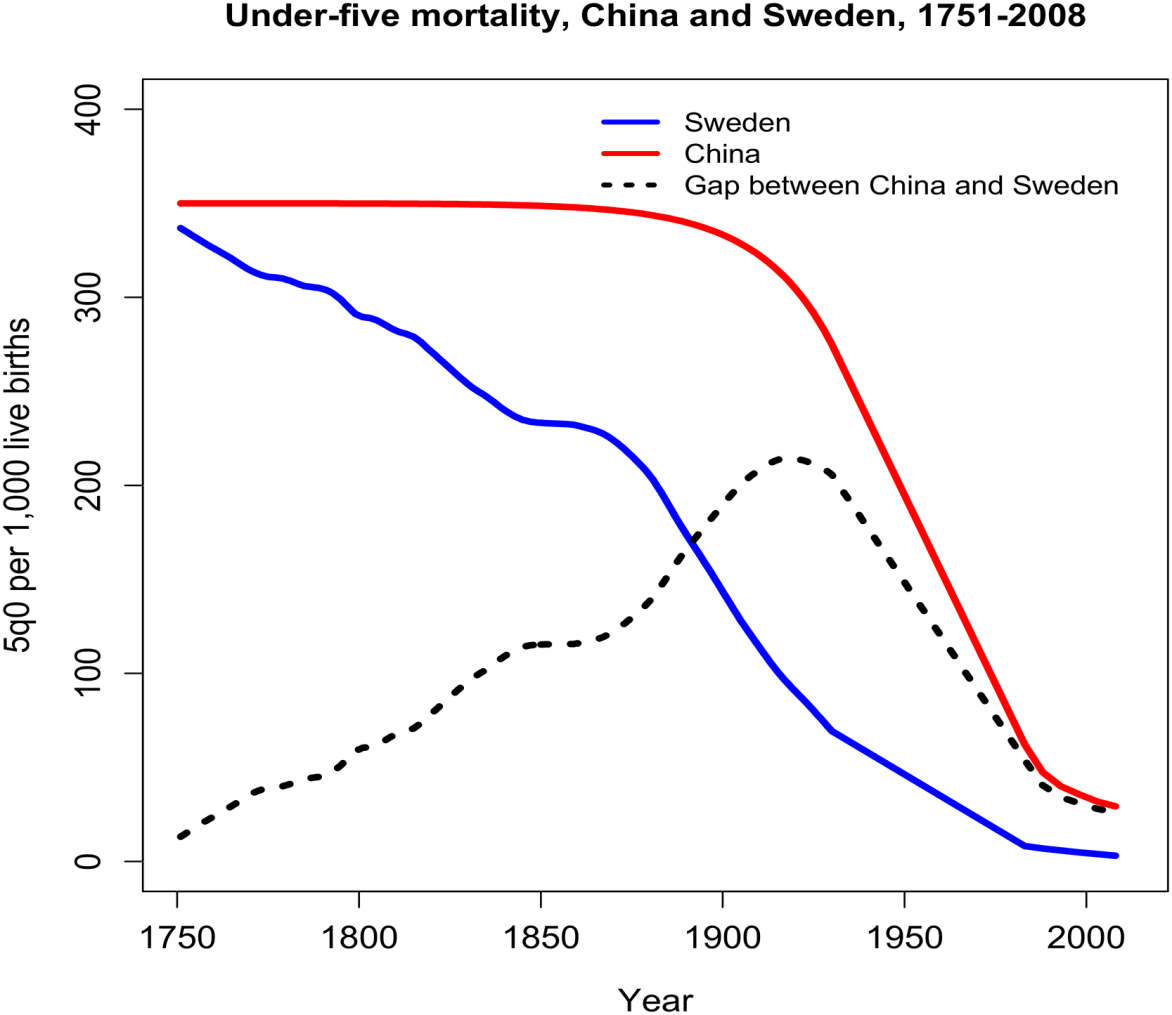
Example of a “high-performing” country overcoming “divergence” in health. Although starting rates of childhood mortality in Sweden and China were comparable in the mid-18^th^ century, Sweden was more successful than China in reducing its level of childhood mortality over time. By the early 20^th^ century, the divergence in child mortality outcomes between the two countries was striking. However, over the next century China’s child mortality rate fell sharply and converged on the Swedish rate, primarily due to China’s scale up of effective health interventions and strengthening of health systems.

The commission’s report “Global Health 2035” found that in less than a generation a significant global convergence could be realized [4]. With effective scale up of proven health interventions, strengthening of health systems, and sustained investment in innovation, by 2035 infectious, maternal, and child deaths rates in low-income countries (LICs) and lower-middle income countries (LMICs) could fall to levels seen today in high-performing middle-income countries. Examples of such “high performers” include the “4-C” middle-income countries, Chile, China, Costa Rica, and Cuba (Fig 1). Moreover, the average incremental cost of this investment in health was estimated at $64B to $83B per year in 2016–2025 and 2026–2035 respectively.

The *Global Health 2035* analysis was conducted independently of the current UN process for drafting the new set of post-2015 Sustainable Development Goals (SDGs) [17]. The analysis has been presented to members of the Open Working Group who are drafting the SDGs. The first draft of the SDGs has now been published, and will be presented and debated at the upcoming UN General Assembly meeting in September 2015. The SDGs will have a target date of 2030, not 2035. In this new study, we therefore estimated what the grand convergence investment case might achieve by 2030, and what level of investment would be required in that time period.

## Methods

This analysis builds on previous work by Stenberg and colleagues for a Global Investment Framework for Women’s and Children’s Health (WCH) [12] and also extends the analysis presented in Jamison et al (2013) to estimate the investment required for grand convergence, as described above. We start by using health outcome and incremental costs for the reproductive, maternal, newborn, and child health (RMNCH) investment case generated by the UN interagency OneHealth Tool (OHT, http://www.avenirhealth.org/software-onehealth) and then we add to this estimated investment in (i) health system strengthening (HSS) to increase the absorptive capacity of local systems to deliver these interventions at scale; (ii) research and development (R&D) costs to develop new medicines, vaccines, diagnostics, and other health technologies; and (iii) the potential costs of scaling up these new health interventions, and (iv) neglected tropical diseases (NTDs).

Our integrated investment framework starts with a country-based (bottom-up) analysis using the OHT to estimate the impact, resource requirements and costs of scaling up proven RMNCH, and select HIV and malaria interventions in LICs and LMICs. The RMNCH interventions span a broad number of categories, including family planning, antenatal care, labor and delivery care, neonatal and post-delivery interventions, immunization, and tools for treating common causes of childhood illnesses and mortality. The full set of RMNCH interventions considered in this study is listed in S1 Table [18]. While the WCH investment case modeled the scale up of 50 evidence-based interventions for 74 countries, our analysis focuses on a sub-set of 34 LIC countries and three large LMICs (India, Indonesia and Nigeria) which comprise roughly three-quarters of global child and maternal mortality. In a few cases, our country grouping includes those that have recently been reclassified into different income groups since the time of the initial CIH analysis. At the time of our analysis, low-income economies were classified as those with a gross national income (GNI) per capita of less than US$1035. Lower-middle income economies had a GNI per capita between US$1036 and US$4085. The countries evaluated in this study are listed in S2 Table.

The analysis was conducted using OHT, version 3.18 Beta 8, a software product whose development is overseen by the UN Inter Agency Working Group on costing, and carried out by the Futures Institute [19]. OHT incorporates previously used epidemiological models, such as the Lives Saved Tool (LiST), the AIDS Impact Model for HIV/AIDS interventions and the FamPlan model to estimate total resource requirements and impact associated with scaling up interventions over time. A key feature of the OHT is that is uses the LiST model as an input to attribute the impact of mortality when simultaneously scaling up multiple interventions, without double counting averted deaths for the same individual. LiST is a critical input to OHT as it follows a set of standard rules that first attributes impact to all preventative interventions, followed by impact due to curative interventions. Interventions are sequentially ordered from pre-conception, through all stages of delivery and for specific age groups including pregnant women 15–49 years of age and age groups for children up to 59 months of age. LiST also uses algorithms to estimate the impact on the same cause of death when there are two or more interventions for prevention or treatment [20]. One of the drawbacks of relying on previously developed models, such as LiST, is that they do not model the impact for all interventions and population target groups. OHT did not capture impact and resource use for all TB interventions, some adult HIV interventions, neglected tropical diseases (NTDs) health system strengthening, and research and development (R&D). In order to capture these investment costs, we incorporated additional estimates of costs to the RMNCH output, using several approaches depending on data availability at the country level. We describe these in greater detail below.

Our projection used the OHT model, data and assumptions applied in the WCH investment case, and compared the most ambitious scale-up scenario, “High” and its least ambitious, “Low (baseline)”. In the “High” scenario in the WCH report, RMNCH interventions are scaled up based on accelerating current trends using a “best performer” approach. The projected trends in coverage for each intervention in a given country were based on the fastest rate of positive change achieved historically since year 2000 in other nations with comparable starting coverage levels. These results (hereafter referred to as the “enhanced investment” scenario) were then compared to the “Low (baseline) scenario, where coverage rates of proven interventions change little over time (hereafter referred to as the “constant coverage” scenario). For each country, we ran the OHT for the set of health interventions listed in S1 Table, scaling up coverage from starting levels in 2011 to higher levels in 2030, based on the best-performer analysis. We estimate incremental costs for scaling up these interventions and the health outcomes they produce from 2012 through 2030 as point estimates for years 2015, 2020, 2025, and 2030. The software models the health effects and resource use for the scaled up scenario. We then used WHO-CHOICE unit cost estimates to estimate the associated investment in commodities and direct system costs required for these RMNCH interventions.

For this analysis, we made a number of minor adjustments to the WCH investment case (and OHT model output) to either reflect new information on health outcomes, such as stillbirths, or to include the impact of malaria and HIV interventions that include either additional interventions or other target population groups, such as both adults and children. These adjustments were essential to capture full impact and to avoid double counting for interventions delivered to a single beneficiary where costs were initially modeled both as part of a population-wide program scale-up and also in a targeted program (e.g., malaria treatment for both pregnant women and all adults). For stillbirths, we adjusted the baseline number of stillbirths projected by the OHT to reflect more recent estimates from the World Health Organization and Save the Children [21]. More recent published estimates of the rate of decline in stillbirths were then applied to each country’s OHT projections to derive a revised number of stillbirths in each scenario over time.

For malaria, a set of interventions for women and children, including malaria treatment, long-lasting insecticide treated nets (LLINs), and intermittent preventive treatment in pregnancy, were included in the WCH scenarios. However, we made three adjustments to the WCH intervention coverage and cost assumptions to extend these interventions and match target coverage rates recommended by the Roll Back Malaria (RBM) Global Malaria Action Plan [6]. First, we extended the coverage of select malaria interventions—for artemisinin-based combination therapies (ACTs) and insecticide treated bednets—from pregnant women and children to the full population at risk [22]. Second, we revised coverage levels of all interventions to match the higher recommended RBM global target coverage levels. These new assumptions were modeled in OHT to produce new country estimates for mortality and cost. Third, we made adjustments to capture the costs of increasing LLIN coverage. We applied a cost of US$5.09 per bednet delivered, including service delivery costs, based on recently published data [23]. We also applied these unit costs to the full population in need, adjusted for the duration of LLIN coverage and for the number of people who would use a bednet, given that some bednets are shared.

For HIV, similar to malaria, a number of interventions were already included in the RMNCH assumptions, such as prevention of mother-to-child transmission (PMTCT), cotrimoxazole, and antiretroviral therapy (ART) for children and pregnant women. Our analysis made three main adjustments to the RMNCH estimates (1) to include the broader adult population in coverage and cost estimates; (2) to reflect higher rates of intervention coverage consistent with an “enhanced investment framework” for HIV/AIDS, reflecting new guidelines aimed at starting ART coverage among patients with higher CD4 counts [24]; and (3) to incorporate the costs and benefits of additional preventative interventions and critical enablers. To extend the scale-up of essential HIV interventions to the broader population and incorporate aspirations of higher-coverage levels, we adapted the recent UNAIDS HIV investment framework [5], modifying it such that ART coverage was projected to reach 80% by 2015, and rise to 90% by 2025, where it would remain until 2030. PMTCT coverage would reach 90% starting in 2015. We also included estimates of the costs and impacts of a number of preventative interventions and critical enablers recommended in the investment framework.

As mentioned above, at the time of our analysis, the module for TB in the OHT did not accurately capture very long-term projections of disease incidence in a context of significantly scaled up interventions. As a result, the TB module in OHT was used only to establish a starting point for current rates of TB in a country, and to estimate the impact of scaled-up HIV interventions on HIV and TB co-infected population. A separate top-down calculation was made of the projected overall decline in TB incidence and mortality, based on analyses and forecasts provided by WHO’s Global TB Programme. These global assumptions were allocated uniformly to each country in the projection.

The potential improvement in TB control under the accelerated cases was based on two assumptions: (1) that incidence rates for TB could decline at rates comparable to the historically best performing regions, and (2) with increases in health coverage, case fatality rates (CFRs) in developing countries could decline over time to rates comparable to those that exist in richer countries (approximately 6%) by 2035. The decline in incidence of TB was projected to change from its current global rate of approximately 2% annually to a 10% rate of decline from 2015 to 2020, a rate of decline experienced in Europe in the post-World War II period. Starting in 2021, the rate of decline was projected to change to 4%, a rate experienced by more recent strong performers such as China [25]. In the Global Health 2035 report, TB rates were then projected to fall to a 2% annual rate of decline starting in 2031. The CFR was projected to decline in straight-line fashion from the country’s starting point to 6% in 2035. To avoid double-counting, the HIV module in OHT was used to forecast the number of deaths in HIV-positive TB patients that would be averted with the scale-up of ART in these countries [18]. The cost of TB interventions was estimated at US$693 per case, based on input from the WHO’s Global TB Programme.

### Health systems strengthening (HSS) investments

Investing in broader health system structures and functions is essential to enable the scaling up of RMNCH, HIV/AIDS, TB, and malaria interventions to the high coverage levels projected in the enhanced investment scenario. To estimate the incremental health system investments required to support such a dramatic increase in coverage levels, we used data from the 2009 Report of the High-Level Taskforce on Innovative International Financing for Health Systems [16].

The Taskforce’s analysis estimated the investments needed to strengthen program management, human resources, infrastructure, equipment and transport, logistics, health information systems, governance and health financing in lower income countries to enable them to meet the MDG targets by 2015. Since the OHT estimates some health system and infrastructure costs related to specific programs, we removed overlapping cost categories from the Taskforce estimates. These costs were then projected to begin in 2013 and extend for a seven-year period, at which point they would stabilize at the final year level. The costs were set to US$2011 dollars, and a real discount rate of 3% was applied to the human resources costs in these categories to reflect the increasing investment required to support the health work force over time. We assumed that 80% of the total projected HSS costs would be allocated to the convergence investment case, while the remainder would be allocated to health system costs for injuries, chronic disease and a number of other conditions not covered in our analysis.

The Taskforce analysis focused on 49 LICs, and did not provide costs estimates for LMICs. For LMICs, which generally spend more on health than LICs, we assumed the need for incremental per capita HSS investments to achieve convergence would be less than the amount needed in LICs. To estimate the fraction of per-capita HSS costs projected for the LICs that would be borne by the LMICs, we compared the current health system spending per capita in LICs and LMICs, and assumed that the remaining per capita HSS investment for LMIC would be 31%, based on the inverse ratio of the current spending on health in these country groupings [4].

### Research and development (R&D) costs and impact

The analyses mentioned so far measure the costs of scaling up existing interventions. However, the 20-year period between 2011 and 2030 could also produce a number of new interventions with potential to impact the burden of these health conditions. To calculate the R&D investments required to fuel this innovation, and the costs and impact of scaling up the new innovations that could emerge from these investments in R&D, a new scenario was created. This “enhanced investment scenario with R&D” projects an incremental reduction in mortality rates from new tools, as well as an incremental cost to invent, procure and deploy those tools.

The costs of investments in R&D for lower-income economies are estimated at US$3 billion per year, based on the recommendations of the Consultative Expert Working Group on Research and Development [11]. This incremental investment would double global R&D investment in this domain to US$6 billion per year. These costs are not allocated to specific countries in our model but are counted at the global level [11].

To estimate the potential reduction in burden driven by newly-invented tools, we drew on research by Jamison, et al., which showed that national adoption of new technologies is associated with a decrease in the under-5 mortality rate of 2% per year [26]. We applied this 2% rate as an accelerator to the average annual decline in under-5 mortality and maternal mortality rates for the LIC and LMIC analyses. In addition, we assumed a 2% incremental decline in the total levels of stillbirths and deaths from HIV/AIDS or TB due to innovation. To estimate the costs of scaling up new health technologies, we assumed that these innovations would perform comparably to existing tools, and therefore would have a cost-benefit ratio equal to existing tools in aggregate. An incremental-cost-per-death-averted was calculated for the enhanced investment scenario compared to the constant coverage scenario. This cost-per-death-averted was then multiplied against the incremental number of lives saved from innovation under the enhanced investment scenario with R&D to estimate the added costs of scaling up the new interventions. This estimate of costs and impacts of new innovation was performed for the years 2015, 2020, 2025, and 2030.

### Neglected tropical diseases (NTDs)

The costs and impact of scaling up interventions to eliminate five NTDs was also added as a separate global cost. This analysis draws on a WHO Africa regional strategy developed in 2012, covering onchocerciasis, lymphatic filariasis, schistosomiasis, blinding trachoma and soil-transmitted helminthes [27]. These are the five NTDs that are amenable to very low-cost mass drug administration. This projection is based on a secondary data analysis that takes a stepwise approach to estimate target population growth and forecasting of inputs and programmatic activities to achieve control and elimination by 2040. The details of this analysis are in Appendix 4 of Global Health 2035 and in a Working Paper by Seddoh et al [18, 28].

In modeling NTD elimination, Seddoh et al [28] estimated that five NTDs could be eliminated with mass drug administration at an annual cost of about US$300–400 million up to about 2020. The cost would then begin to fall as transmission is interrupted and as the burden falls to a level that can be managed by the public health system. Much of the burden of NTDs (90%) in sub-Saharan Africa is from these five diseases.

### Integration of analyses

Several final adjustments were made in the model to integrate assumptions across health conditions and to extend the projections to 2030. We applied a real discount rate of 3% to the WHO-CHOICE unit costs (excluding commodities) to capture the potential inflation in health system capacity investments needed to expand the workforce to deliver these interventions. To capture the impact and costs of scaling up health interventions for LMICs, we extrapolated estimates from India, Indonesia and Nigeria, the three largest LMICs that account for more than 70% of the population in LMICs, and nearly two-thirds of the births. Like many investment analyses, uncertainty is introduced into the model through input values and the underlying structural assumptions. We conduct scenario analysis to provide results for plausible alternative scenarios, as recommended by ISPOR task force report for conducting budget impact analysis [29]. Results are presented for a conservative constant coverage scenario and an enhanced investment scenario.

**Fig 2:**
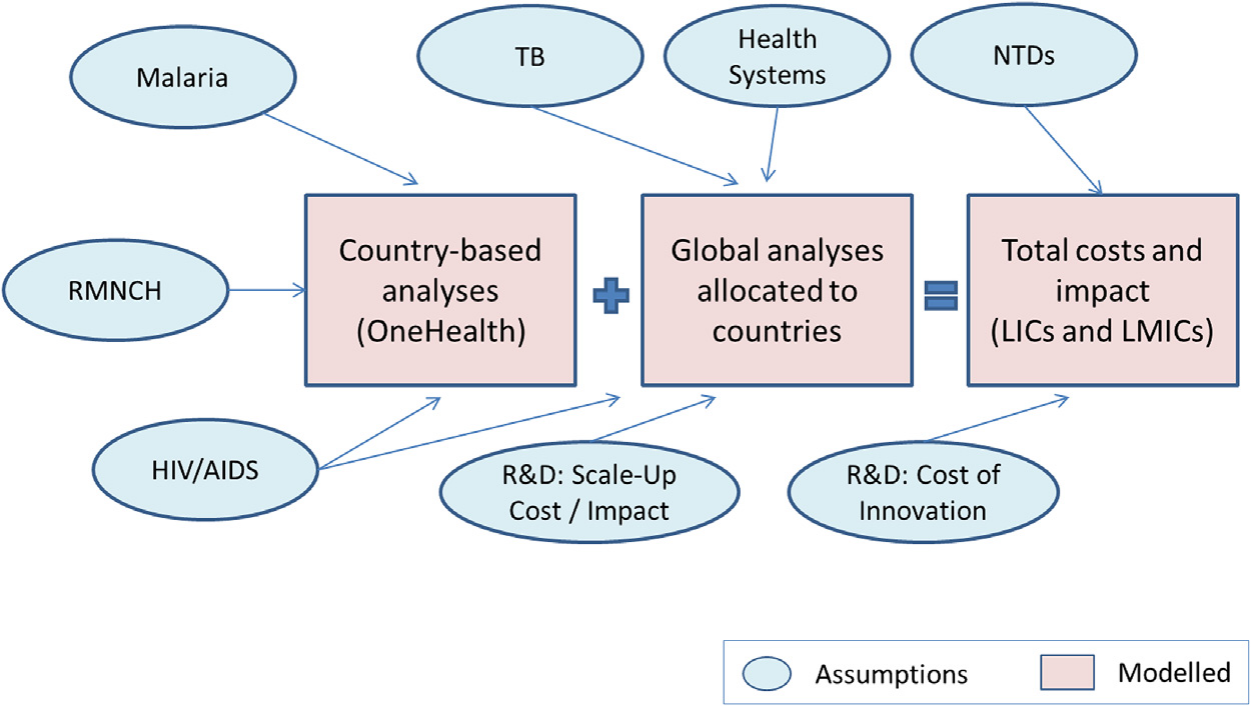
Convergence analysis structure.

To arrive at the total convergence cost for 2015 to 2030, we aggregated country level program specific impact and cost data for several years (2015, 2020, 2025, and 2030). These amounts were calculated by World Bank income classifications for LICs and LMICs. The calculated results for each country segment were then rounded to avoid false precision in the forecasts and to provide a more general target for the potential impact and cost of each scenario. Fig 2 summarizes the overall approach used to calculate each portion of the analysis described above.

## Results

### Low income countries

Investments to scale up evidence-based interventions in 34 low-income countries would result in a major reduction in mortality (Table 1). In the year 2030, the enhanced investment scenario with R&D would avert more than 6 million deaths compared to the constant-coverage scenario in these 34 low-income countries.

**Table 1:** Impact of the grand convergence investment case on low-income countries*.

	Baseline 2011	Constant coverage scenario 2030	Enhanced investment scenario 2030	Enhanced investment scenario with R&D 2030	Events averted by enhanced investment in 2030
	A	B	C	D	B-D
**RMNCH**					
Births, thousands	27,473	43,500	23,000	23,000	20,500
Total Fertility Rate	4.2	4.3	2.3	2.3	
Maternal deaths, thousands	113	188	41	27	160
Stillbirths, thousands	736	1150	400	275	875
Neonatal deaths (0–27 days), thousands	897	1450	220	150	1300
Child deaths (1–59 months), thousands	1969	3300	700	475	2825
Total Under 5 Child deaths, thousands	2866	4750	925	625	4125
Under-5 mortality Rate, deaths per 1000 births	104	109	40	27	82
Maternal Mortality Rate, deaths per 100,000 births	412	431	178	119	312
**Tuberculosis**					
New cases, thousands	2010	2050	1000	675	1375
Deaths, thousands	430	440	100	60	380
**HIV/AIDS**					
New infections, thousands	858	1,400	200	135	1265
Deaths aged 5 and over, thousands	598	950	100	70	880
Total HIV/AIDS & TB deaths, thousands	892	1200	175	125	1075
**Total deaths, thousands**	**4606**	**7300**	**1550**	**1050**	**6250**

*Data presented are rounded.

Increasing uptake of proven interventions yields the largest change in mortality, and technical progress further enhances the impact of the investment scenarios. Under-5 mortality in these countries declines from an estimated 104 deaths per 1000 live births in 2011 to 40 per 1000 live births in 2030 simply from the increase in coverage of existing interventions. The addition of innovative new tools leads the under-5 mortality rate to decline further to 27 deaths per 1000 live births. A comparable effect is seen in the maternal mortality rate. Maternal deaths decline from 412 per 100,000 births in 2011 to 178 per 100,000 births in 2030 from expanding coverage of existing interventions. The rate declines further to 119 maternal deaths per 100,000 live births with the addition of assumptions around technical progress and new tools [1]. According to the projections, there would be more than 1 million fewer deaths from HIV and TB in 2030 in the enhanced investment scenario with R&D than in the constant coverage scenario.

Table 2 lays out the annual incremental investment requirements for LICs in 2015, 2020, 2025, and 2030. Over this 15 year period, the cumulative incremental investment above the constant-coverage scenario in for LICs would be around $370 billion. In 2015, the incremental costs would be $24.3 billion, including a relatively large component of HSS costs as the interventions are scaled up. The incremental costs would decline slightly by 2020 to $21.8 billion as the completion of some HSS investments more than offsets increased spending on programmatic costs. The costs would then rise to $24.7 billion in 2025 and $27 billion in 2030 to reflect increases in population, intervention coverage, and the effects of inflation. These data translate into an incremental cost per capita between US$26 and US$21, depending on the year [18]. In this analysis, global costs for investments in R&D to invent new interventions are excluded.

**Table 2:** Annual incremental costs of the grand convergence investment case in low-income countries, excluding R&D* ^ⱡ^.

	Incremental Costs			
US$ Million	2015	2020	2025	2030
**Programmatic Investment (Scale Up: Current Tools)**				
Family Planning	125	300	450	650
Maternal and Newborn health	200	500	800	1,100
Immunization	625	700	675	700
Treatment of Childhood illness	325	625	750	650
Malaria	1,350	1,575	1,825	2,150
Tuberculosis	1,050	675	675	650
HIV/AIDS	1,100	2,000	2,825	3,625
Subtotal	4,800	6,400	8,000	9,500
**Health System Strengthening**				
Incremental investment	17,500	13,400	14,700	15,500
**Programmatic Investment (Scale Up: New Tools)**				
All new tools and interventions	2,000	2,000	2,000	2,000
**Total Investment**	**24,300**	**21,800**	**24,700**	**27,000**
**Ratios**				
Population (M)	934	1,024	1,178	1,251
Incremental cost per capita ($)	26	21	21	22

*Data presented are rounded.

^**ⱡ**^ Excludes cost of incremental global R&D, which is estimated at $3B annually.

Direct programmatic costs for existing interventions under the enhanced investment scenario with R&D comprise slightly around one-third of the annual incremental costs. The composition of these costs shifts over time and across countries, with program costs rising in the later years after some of the heavier investments in early years in HSS have been completed. HSS is the biggest driver of overall incremental costs, comprising more than 70% of costs in 2015 and falling to 56% in 2030. Of these HSS costs, the dominant component is infrastructure, including equipment and vehicles. An important outcome from such investments is that it leads to a *functional health system platform for service delivery* that can tackle other long-term health challenges, not just infections and RMNCH conditions. The cost of scaling up the new health interventions generated by investments in R&D is estimated at an additional incremental cost of $2.5-$3.5B per year for low-income economies, or about 10–15% of the total cost, depending on the year. These costs are summarized in Table 2.

From a cost-benefit standpoint, the projected enhanced investment scenario with R&D would represent a highly attractive investment. The cost per death averted in this scenario starts at US$11,600 in 2015 (the time of maximum investment in HSS and before the interventions are fully scaled up) and declines to US$4,300 by 2030. Using a full income approach to estimating the economic benefits of convergence (described in Appendix 3 of “Global Health 2035”, at http://globalhealth2035.org) [18], benefits would exceed costs by a factor of about 9.

### Lower-middle income countries

In the 48 LMICs, there would also be a sizable reduction in mortality from the scale up of proven health interventions (Table 3). In 2030, the enhanced investment case with R&D would avert more than 6.6 million deaths in LMICs compared to the constant-coverage scenario.

**Table 3:** Impact of the grand convergence investment case on lower-middle-income countries*.

	Baseline 2011	Constant coverage scenario 2030	Enhanced investment scenario 2030	Enhanced investment scenario with R&D 2030	Events averted by enhanced investment in 2030
	A	B	C	D	B-D
**RMNCH**					
Births, thousands	60,168	71,500	52,250	52,250	19,250
Total Fertility Rate	2.9	2.9	2.1	2.1	
Maternal deaths, thousands	156	200	54	36	167
Stillbirths, thousands	1331	1675	700	475	1200
Neonatal deaths (0–27 days), thousands	1750	2125	425	275	1850
Child deaths (1–59 months), thousands	2041	2650	625	425	2225
Total Under 5 Child deaths, thousands	3791	4775	1050	700	4075
Under-5 mortality Rate, deaths per 1000 births	63	67	20	13	54
Maternal Mortality Rate, deaths per 100,000 births	260	284	104	69	215
**Tuberculosis**					
New cases, thousands	4,287	3875	1900	1275	2600
Deaths, thousands	705	650	150	100	550
**HIV/AIDS**					
New infections, thousands	723	1025	150	100	925
Deaths aged 5 and over, thousands	568	775	75	50	725
Total HIV/AIDS & TB deaths, thousands	1164	1300	200	125	1175
**Total deaths, thousands**	**6,442**	**7950**	**2000**	**1350**	**6600**

*Data presented are rounded.

As in the LICs, the development and application of new tools could potentially increase the number of deaths averted in LMICs by several million more over this timeframe. In the enhanced investment scenario with R&D, under-5 mortality would fall from 67 deaths per 1000 live births in 2011 to 13 in 2030, including the impact from new tools; maternal mortality rates would decline from 260 deaths per 100,000 births in 2011 to 69 per 100,000 births in these countries by 2030. The results from these scenarios in 2030 are summarized in Table 3.

In lower-middle-income economies, the enhanced investment case with R&D would carry a cost of $670 billion over the 15-year period between 2016 and 2030. The estimated incremental costs would be US$34.75 billion per year in 2015, and would rise to $38.9 billion, $48.7 billion, and $56.3 billion respectively in 2020, 2025, and 2030. This amounts to an incremental annual cost per capita of about US$14 in 2015 rising to $19 in 2030 in the LMICs (Table 4).

**Table 4:** Annual incremental costs of the grand convergence investment case in lower-middle-income countries* ^ⱡ^.

	Incremental Costs			
US$ Million	2015	2020	2025	2030
**Programmatic Investment (Scale Up: Current Tools)**				
Family Planning	400	750	1,200	1,700
Maternal and Newborn health	1,800	3,500	5,400	7,200
Immunization	1,000	3,500	4,300	4,900
Treatment of Childhood illness	1,300	3,800	6,000	6,400
Malaria	4,650	5,700	6,900	8,400
Tuberculosis	2,450	1,650	1,600	1,600
HIV/AIDS	1,000	2,200	3,500	4,800
Subtotal	12,600	21,100	28,900	35,000
**Health System Strengthening**				
Incremental investment	18,650	13,800	14,800	15,300
**Programmatic Investment (Scale Up: New Tools)**				
All new tools and interventions	3,500	4,000	5,000	6,000
**Total Investment**	**34,750**	**38,900**	**48,700**	**56,300**
**Ratios**				
Population (M)	2,535	2,684	2,828	2,962
Incremental cost per capita ($)	14	14	17	19

*Data presented are rounded.

^**ⱡ**^ Excludes cost of incremental global R&D, which is estimated at $3B annually.

In contrast to the LICs, the majority of the incremental costs in the LMICs are for programmatic scale-up of existing interventions. Aside from 2015, when the HSS investments are largest and the programmatic interventions are still being scaled up, programmatic investments make up more than half the total incremental investment in LMICs (and nearly two-thirds by 2030). This difference between the LICs and LMICs is attributable to the higher level of per capita health spending today in LMICs, which suggests that less investment is needed in HSS than in the LICs to achieve a robust scale up of the interventions modeled. The cost per death averted in LMICs is also larger than the cost per death averted estimated for LICs. It declines from US US$11,600 in 2015 to US US$8,500 in 2030. Using a full income approach to estimating the economic benefits, the benefits would exceed costs by a factor of more than 15.

The total impact and costs for both LIC and LMIC are presented in Tables 5 and 6, respectively. They show that the enhanced investment scenario with R&D would yield nearly 13 million fewer deaths in 2030 in LICs and LMICs than the constant-coverage scenario, with incremental annual costs rising from US$62 billion in 2015 to $86 billion in 2030.

**Table 5:** Impact of the grand convergence investment case on low and middle income countries*.

	Baseline 2011	Constant coverage scenario 2030	Enhanced investment scenario 2030	Enhanced investment scenario with R&D 2030	Events averted by enhanced investment in 2030
	A	B	C	D	B-D
**RMNCH**					
Births, thousands	87,641	115,000	75,250	75,250	39,750
Total Fertility Rate	3.2	3.3	2.2	2.2	
Maternal deaths, thousands	269	388	95	63	325
Stillbirths, thousands	2,067	2,825	1,100	750	2,075
Neonatal deaths (0–27 days), thousands	2,647	3,575	645	425	3,150
Child deaths (1–59 months), thousands	4,010	5,950	1,325	900	5,050
Total Under 5 Child deaths, thousands	6,657	9,525	1,975	1,325	8,200
Under-5 mortality Rate, deaths per 1000 births	76	83	26	18	65
Maternal Mortality Rate, deaths per 100,000 births	307	337	126	84	254
**Tuberculosis**					
New cases, thousands	6,297	5,925	2,900	1,950	3,975
Deaths, thousands	1,135	1,090	250	160	930
New infections, thousands	1,581	2,425	350	235	2,190
Deaths aged 5 and over, thousands	1,166	1,725	175	120	1,605
Total HIV/AIDS & TB deaths, thousands	2,056	2,500	375	250	2,250
**Total deaths, thousands**	11,049	15,238	3,540	2,388	12,850

*Data presented are rounded.

**Table 6:** Annual incremental costs of the grand convergence investment case in low and middle income countries*.

	Incremental Costs			
US$ Million	2015	2020	2025	2030
**Programmatic Investment (Scale Up: Current Tools)**				
Family Planning	525	1050	1,650	2,350
Maternal and Newborn health	2000	4000	6,200	8,300
Immunization	1625	4200	4,975	5,600
Treatment of Childhood illness	1625	4425	6,750	7,050
Malaria	6000	7275	8,725	10,550
Tuberculosis	3500	2325	2,275	2,250
HIV/AIDS	2100	4200	6,325	8,425
Subtotal	17,375	27,475	36,900	44,525
**Health System Strengthening**				
Incremental investment	36,150	27,200	29,500	30,800
**R&D for New Tools**				
Incremental Investment	3,000	3,000	3,000	3,000
**Programmatic Investment (Scale Up: New Tools)**				
All new tools and interventions	5,500	6,000	7,000	8,000
**Total Investment**	**62,025**	**63,675**	**76,400**	**86,325**
**Ratios**				
Population (M)	3,469	3,708	4,006	4,213
Incremental cost per capita ($)	18	17	19	20

*Data presented are rounded.

**Fig 3:**
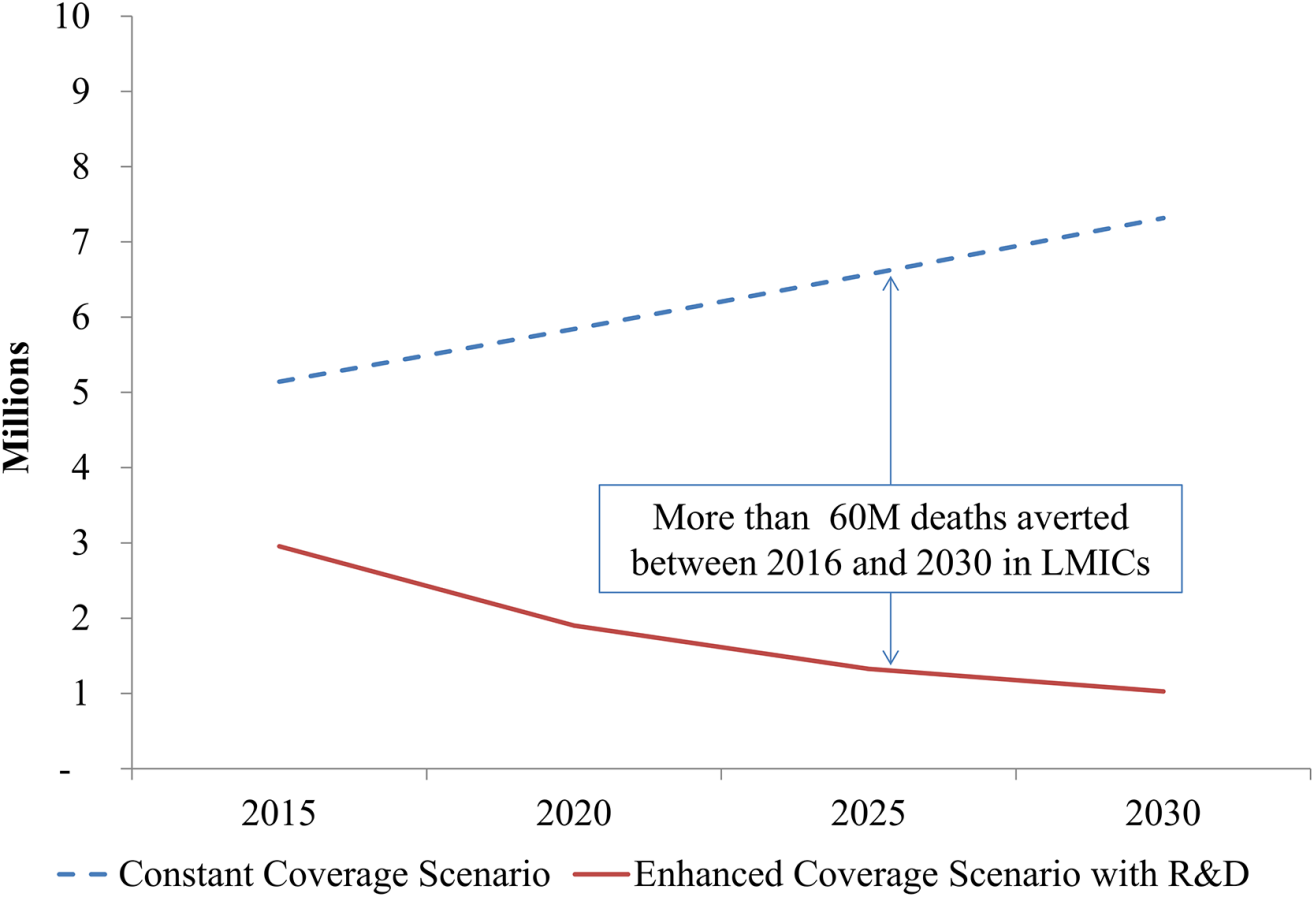
Deaths per year across scenarios, low-income countries. 2016–2030.

**Fig 4:**
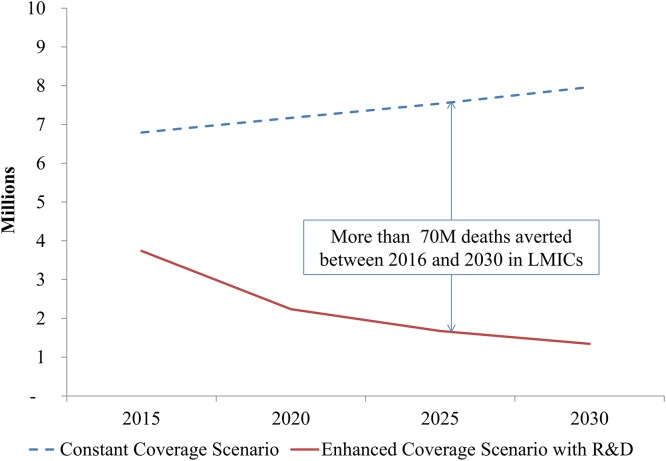
Deaths per year across scenarios, low-middle income countries. 2016–2030.

Altogether, the enhanced investment scenario with R&D could avert a significant number of deaths over the course of the investment period compared to the constant coverage scenario. Figs 3 and 4 compare the total number of modeled deaths per year in LICs and LMICs, respectively. In LICs, the investments to scale up critical interventions, strengthen health systems, and implement new tools would avert more than 60 million deaths (including stillbirths) over this 19-year period compared to the scenario where coverage is maintained at current levels. In LMICs, the total number of deaths averted would be more than 70M over this time period. Roughly two-thirds of these deaths averted would be children under 5.

## Discussion

This analysis suggests that key health outcomes in LICs and LMICs can converge to a significant degree with those of wealthier countries by 2030, and that the notion of a “grand convergence” may serve as a unifying theme for health indicators in the SDGs. Success in scaling up health interventions at rates achieved in recent years by other countries (e.g., Rwanda, Turkey) would dramatically reduce the burden of disease in even the poorest countries. In our enhanced investment scenario with R&D, the scale up of current and new tools will reduce the under-5 mortality rate (U5MR) from 104 per 1000 live births to 27 per 1000 live births in LICs, and from 63 per 1000 live births to 13 per 1000 live births in LMICs by 2030. While this improvement does not bring the poorest countries all the way to OECD levels, it compares well to upper-middle-income countries, which were estimated by the World Bank to have a U5MR of 20 in 2012.

The convergence analysis projects dramatic improvements in other key health indicators, including the maternal mortality rate, the number of stillbirths, and the number of deaths from HIV/AIDS and TB. In all these conditions, mortality falls sharply. In 2030 alone, nearly 13 million deaths occur in the enhanced investment scenario with R&D than in the constant-coverage scenario; the deaths averted are split nearly evenly between LICs and LMICs. These figures include maternal and young child deaths averted, stillbirths averted, and reductions in over-5 HIV and TB deaths.

The scale-up of health interventions also has a profound effect on the demographics of these countries. In addition to saving a large number of young lives, the enhanced investment scenario with R&D calls for a rapid scale-up of family planning interventions in high-fertility countries. The expansion of family planning tools greatly reduces the number of unwanted pregnancies in these countries, and causes the total fertility rate to fall from 4.2 to 2.3 in LICs, and from 2.9 to 2.1 in LMICs. The birth rates in these countries decline sharply, and the projected total number of births in 2030 remains lower than 2011 levels, despite an increase in overall population from growing life expectancies. This demographic change promises a number of potential benefits for these countries, including reducing the total costs of health interventions targeting mothers and young children (e.g., labor and delivery, newborn care, immunization), reducing the burden on health systems, creating incentives for investments in education and in women, and yielding a potential economic demographic dividend.

Implementing the convergence scenario would require a substantial increase in health investment. The price tag for LICs is an additional US$22–27 billion per year from 2016 to 2030. For LMICs, the annual incremental costs would rise from US$35 billion in 2016 to $56 billion in 2030. In addition, we forecast the need for an incremental increase of US$3B globally for R&D to develop new health interventions for poorer countries, along with a US$300–400 million for NTDs in the first decade of the enhanced investment scenario with R&D. Altogether, that means an average annual incremental cost of ~US$62 billion in 2015, rising to US$86 billion in 2030 (Table 6).

This projected rise in annual investment costs is driven by a number of factors. One factor is the direct costs (commodity and provider) associated with the increased coverage levels of these essential interventions over a growing population. A second, and larger, factor is the increased investment in the underlying health system needed to support and sustain this expansion of health care in these countries. Finally, there is the impact of our assumption that wages and system costs are elastic and will rise over time as the health systems grow in scale and scope. Any of these assumptions may be overstated. If real salaries do not rise as sharply in practice as predicted or are offset through innovations in delivery systems, the true costs in the out-years of our forecast may be smaller than our estimates. In addition, improvements in targeting of interventions or efficiencies in the design and implementation of health systems may allow for further reductions in costs compared to our estimates.

The overall cost figure for achieving convergence may seem at first to be beyond reach. Current investments in development assistance for health (DAH) have been estimated at less US$30B in 2011, with year-on-year growth slowing after a decade of rapid increases [30]. However, DAH is only one source of funding for convergence, and domestic financing may provide additional resources to close the gap. The IMF estimates that public spending on health is about 2% of GDP for LICs and 1·7% of GDP for LMICs [4]. It also estimates that, as a result of anticipated economic growth, LICs are on course to add almost US$1 trillion per year to their GDP from 2016 to 2030, and LMICs are projected to add almost US$9 trillion per year in the same time period [4]. If LICs and LMICs were to increase domestic spending to 3–4% of GDP, given the projected GDP growth, the cost of convergence in many countries could be funded domestically.

Evaluating the convergence investment on a per-capita basis provides a different and seemingly more affordable perspective. In terms of incremental cost per capita, the enhanced investment scenario with R&D would cost about US$21–26 and US$14–19 more per person per year in LICs and LMICs, respectively. Based on estimates presented in Tables 1 and 2, the estimated incremental cost per death averted averages around US$5000 for most of the projection timeframe in LICs. In LMICs, it averages around US$8000 in incremental costs to avert a death. Most of the deaths averted in LICs and LMICs from the convergence investment are for children under 5, whose survival provides a larger number of life-years gained than from a death averted in the general population on average.

An integrated scale up strategy also has synergistic effects across interventions. First of all, strengthening health systems to enable the scale up of certain interventions has the potential to create more robust and effective channels for health care delivery at the community, outreach, clinic and hospital level, as a number of policymakers and experts have noted [31]. These improvements hold the promise of improving care not just in vulnerable populations or certain conditions, but also for the broader community, and in non-communicable diseases (NCDs) as well as infections.

Second, there are interdependencies across categories of interventions that make a broad scale-up strategy attractive. For example, accelerating the uptake of family planning interventions reduces unwanted pregnancies and sharply lowers the birth rate in high-fertility countries. This outcome yields a number of positive consequences for health systems: fewer women needing interventions for complicated labor and deliveries, fewer stillbirths, fewer neonatal health complications, and fewer children needing immunization. This reduction in demand means a low-income nation can achieve much higher levels of coverage of key maternal and young child health interventions with only modest increases in investment, and without much additional strain on the absorptive capacity of the health system.

The interdependent relationship between HIV and TB interventions is another good example, as many LICs have very high rates of HIV and TB coinfection; strategies to scale up ART among HIV-positive patients at earlier stages of disease will reduce the prevalence of TB. Similarly, increased use of preventative tools like vaccination will lower the subsequent need for treatment for some categories of childhood illness. While scarcity of financial or human resources may force careful prioritization among otherwise favorable investments, a fully integrated approach that includes a full range of interventions is likely to be less expensive and more effective than the sum of individual investment cases for independent vertical programs.

There are many important caveats to this modeling study. First, all models depend on the accuracy of the assumptions used and their translatability to new settings. Our projection assumes that proven interventions work at a comparable level of efficacy in our modeled countries as in other settings, and with comparable costs. To the extent that conditions on the ground are different in the countries modeled than in other settings, the performance of these interventions may be different. In practice, there is likely to be considerable heterogeneity in the costs and outcomes experienced in different countries, but we are unable to show the range of possible measures of uncertainty due to the limitations of our modeling tools.

We also assume that the starting levels of burden in our models—drawn from UN inter-agency estimates of under-5 mortality rates made in 2010 [32]–serve as an accurate starting baseline from which to project future trends. Our projections of health impact would need to be modified if estimates of the starting burden of disease are updated significantly, or if the effectiveness of modeled interventions is shown to be significantly different from the estimates of effectiveness at the time of the modeling.

Second, there are several caveats to note around our cost estimates. Our estimates are based on current or recent costs in each country modeled, and over the next generation, the costs of procuring and delivering these interventions may well change in unpredictable ways. Investments to rapidly scale up interventions could lower the costs of individual interventions in the form of scale economies in procurement and production. Alternatively, a broad scale up of multiple interventions in parallel could also cause the costs of health care workers and other resources to rise. To the extent that our models fail to capture adequately these cost elasticity and scale effects, we may misstate the costs of convergence. In addition, estimates of costs are based on populations at risk as they stand today. To the extent that health interventions impact disease epidemiology and narrow the target population (e.g., shrinking the map for malaria or other vector-borne diseases), the costs in future years as modeled may overstate the actual costs.

Third, convergence depends on political stability and a health system capable of delivering a broad range of interventions effectively over time. Since the countries modeled include some of the poorest and most troubled countries in the world, this is a legitimate challenge to the aspirations of convergence. We have sought to address this question by including a significant level of investment in health system strengthening and increased the costs of interventions over time to reflect the challenge of expanding the health workforce in these settings. However, to the extent that these investments are insufficient to enable the scale up, our models may overstate the achievable burden reduction or understate the costs of convergence. On the other hand, our analysis of the impact does not account for any potential benefit from these investments in improving care for chronic and non-communicable diseases, which are a growing source of health burden in much of the world. Additional research is needed to understand the differential impact that NCDs will have on human health in different geographies and the cost effectiveness of interventions to achieve closer convergence in outcomes for those conditions.

Fourth, our models focus exclusively on health interventions and do not account for changes exogenous to the health sector. We do not account for the positive impacts on health that may accrue, for example, from investments to improve agricultural productivity and dietary diversity, education (especially for women), water and sanitation systems, quality of housing stock, or other systems with potentially pro-health impacts [33]. At the same time, we also do not account for the negative effects of war and civil strife, climate change, declines in the efficacy or loss of key interventions from resistance, or the emergence of new health threats that would impact these populations. The balance of these factors over time will have a profound impact on overall health and on the scale up of health interventions that we are not able to model.

Fifth, our convergence scenario depends on a continued and expanded investment in health R&D, and assumes that this investment yields improved health outcomes comparable to what historical innovations have delivered. This may not be the case over the next 15–20 years, as there is evidence that innovations in health are becoming harder to find [34]. If rates of innovation are lower (or higher) than historical experience, or if the cost-benefit ratios of those innovations are lesser (or greater) than historical trends, the results will be different from what is in our models. In addition, we have no way of foreseeing in which areas innovation will occur, and so have applied a flat rate of improvement (incremental 2% declines) to all conditions; in practice, breakthrough innovations may strike in some specific areas and not others, and so the actual impact may be more skewed across conditions than our modeling shows.

Finally, the projections included in this modeling work depend on countries and donors taking a holistic and integrated perspective on their health investments. Convergence relies on scaling up a full range of interventions across all affected populations. It is worth noting, therefore, that some of the key beneficiaries of these interventions are marginalized groups with limited political voice (e.g., the poorest citizens, young children, adolescent girls, groups at high-risk for HIV). Some key interventions (e.g., family planning, immunization) may be unpopular or controversial in certain quarters. As a result, countries looking to converge will need to take a comprehensive and equitable distribution of health investments in order to reach those most in need, and ensure resources are allocated in a manner consistent with need. To the extent that countries fail to adopt a mindset towards (a) universal health coverage of publicly-financed interventions for infections and RMNCH conditions, and (b) ensuring the rights of groups that are key to this scale up (e.g., girls and women; men who have sex with men), our models will overstate the benefits.

## Conclusion

A grand convergence in health may be within our reach. Over the next generation, investments to scale up proven health interventions and to develop innovative new tools could reduce mortality rates in even the poorest countries to levels comparable to those in wealthier nations. By 2030, the time horizon for the SDGs, an integrated investment plan for low-income and lower-middle-income countries could yield under-5 mortality rates comparable to upper-middle-income countries today, and set the stage for continued convergence with high-income countries. The stark disparities that exist in health today across countries and communities can be greatly reduced, creating more equal opportunities for survival and well-being everywhere.

While the technical means exist to achieve this grand convergence, success will depend on sustained commitments by international and local actors. Targeted investments in productive development assistance for health must be combined with national efforts that make investing in health a priority. Universal approaches to health care are essential to ensure the poorest communities can access essential elements of care. Programs must also account for the needs of vulnerable and marginalized populations. By embedding a global convergence into the SDGs, we can ensure a sustained commitment to the global and national investments in health that can mark the start of a third—healthier and more equitable—phase in human health.

## Supporting Information

S1 Table List of interventions included in the RMNCH analysis. (DOCX)

S2 Table Low-income countries (LICs) modeled in the investment case*. (DOCX)

S1 Data Raw dataset. (XLSX)
