# Supplementary material for: Achieving a “Grand Convergence” in Global Health: Modeling the Technical Inputs, Costs, and Impacts from 2016 to 2030
Source: PLoS One. 2015 Oct 9;10(10):e0140092. doi: 10.1371/journal.pone.0140092 (PMC4599920; doi:10.1371/journal.pone.0140092)
Supplement: S1 Table — (DOCX) [file pone.0140092.s001.docx]

**S1 Table**

List of interventions included in the RMNCH analysis

| **Category** | **Package** |
| --- | --- |
| **RMNCH** | **Package 1: Family Planning** |
|  | Modern family planning methods (pill, condom, injectable, IUD, implant, female sterilization, male sterilization, lactational amenorrhea method, vaginal barrier method, vaginal tablets, other contraceptives) |
|  | **Package 2: Maternal and Newborn Health** |
|  | Safe abortion |
|  | Post-abortion case management |
|  | Ectopic case management |
|  | Syphilis detection and treatment (pregnant women) |
|  | Multiple micronutrient supplementation |
|  | Balanced energy supplementation |
|  | Management of pre-eclampsia (magnesium sulphate) |
|  | Detection and management of diabetes in pregnancy |
|  | Detection and management of fetal growth restriction |
|  | Labour and delivery management |
|  | Active management of the 3rd stage of labor |
|  | Management of eclampsia (magnesium sulphate) |
|  | Neonatal resuscitation (institutional) |
|  | Kangaroo mother care |
|  | Clean practices and immediate essential newborn care (home) |
|  | Antenatal corticosteroids for preterm labor |
|  | Antibiotics for preterm premature rupture of the membranes (pPRoM) |
|  | Induction of labour (beyond 41 weeks) |
|  | Neonatal infections/newborn sepsis - Full supportive care |
|  | Preventive postnatal care |
|  | Periconceptional folic acid supplementation |
|  | Calcium supplementation for prevention and treatment of pre-eclampsia and eclampsia |
|  | **Package 3: Malaria** |
|  | Malaria treatment (children) |
|  | Insecticide treated materials |
|  | Pregnant women sleeping under an ITN |
|  | Intermittent preventive treatment - IPT (pregnant women) |
|  | Treatment of malaria (pregnant women) |
|  | **Package 4: HIV** |
|  | Prevention of mother to child transmission (PMTCT) |
|  | ART (first-line treatment) for pregnant women |
|  | Cotrimoxazole for children |
|  | Pediatric ART |
|  | **Package 5: Immunization** |
|  | Tetanus toxoid (pregnant women) |
|  | Rotavirus vaccine |
|  | Measles vaccine |
|  | DPT vaccination |
|  | Hib vaccine |
|  | Polio vaccine |
|  | BCG vaccine |
|  | Pneumococcal vaccine |
|  | Meningitis vaccine |
|  | **Package 6 : Child Health** |
|  | Oral rehydration therapy |
|  | Zinc (diarrhea treatment) |
|  | Antibiotics for treatment of dysentery |
|  | Pneumonia treatment (children) |
|  | Vitamin A for measles treatment (children) |
|  | Breastfeeding counseling and support |
|  | Complementary feeding counseling and support |
|  | Management of severe malnutrition (children) |
|  | Management of moderate acute malnutrition |
|  | Vitamin A supplementation in infants and children 6-59 months |

*RMNCH: Reproductive, Maternal, Neonatal, and Child Health*

* In countries where abortion is legal.

** Current analysis includes impact only, not cost.
