# Supplementary material for: Achieving a “Grand Convergence” in Global Health: Modeling the Technical Inputs, Costs, and Impacts from 2016 to 2030
Source: PLoS One. 2015 Oct 9;10(10):e0140092. doi: 10.1371/journal.pone.0140092 (PMC4599920; doi:10.1371/journal.pone.0140092)
Supplement: S2 Table — (DOCX) [file pone.0140092.s002.docx]

**S2 Table**

Low-income countries (LICs) modeled in the investment case*

| Afghanistan | Congo, Dem. Rep | Madagascar | Rwanda |
| --- | --- | --- | --- |
| Bangladesh | Eritrea | Malawi | Sierra Leone |
| Benin | Ethiopia | Mali | Somalia |
| Burkina Faso | Guinea | Mauritania | Tajikistan |
| Burundi | Guinea-Bisau | Mozambique | Tanzania |
| Cambodia | Haiti | Myanmar | Togo |
| Central African Republic | Kenya | Nepal | Uganda |
| Chad | Korea, Dem Rep. | Niger | Zimbabwe |
| Comoros | Kyrgyz Republic |  |  |

*Note that low-middle income countries (LMIC) included were India, Indonesia, and Nigeria. These three countries account for greater than 70% of the total LMIC population. The aggregated figures and trends for these three countries were extrapolated to the rest of the LMIC group based on relevant demographic information.
